# Perception of Simulation and Virtual Reality (VR) in Surgery in the Public Sector Tertiary Care Teaching Hospitals of Southern Punjab, Pakistan: A Cross-Sectional Survey

**DOI:** 10.7759/cureus.79428

**Published:** 2025-02-21

**Authors:** Syed Mustafa Haider, Narmeen Fatima, Muhammad Hassan Abbas

**Affiliations:** 1 Department of General Surgery, Surgical Unit 2, Sheikh Zayed Medical College and Hospital, Rahim Yar Khan, PAK

**Keywords:** benefits, challenges, effectiveness, implementation, pakistan, simulation, southern punjab, virtual reality (vr)

## Abstract

Background: The use of virtual simulation (VS)-based institutional practices is increasing with a gradual shift in the incorporation of the latest technology such as virtual reality (VR) and artificial intelligence (AI) in medical education. Our cross-sectional study aimed to evaluate the perception and practice of simulation and VR among professionals in surgery. It was also aimed to identify how their integration would affect the surgical practice, including their significance and acceptance among relevant stakeholders.

Materials and methods: A pre-designed questionnaire was filled in by 218 participants, from all four public sector tertiary care teaching hospitals of the southern Punjab region of Pakistan. A sample size of 218 was calculated with a 5% margin of error and an 80% confidence interval. The questionnaire included sections on perception, familiarity, and effectiveness of integrating simulation and VR into the surgical practice. The survey was performed from January 1, 2025, to January 31, 2025. Data was collected through online platforms (Google Forms, Google LLC, Mountain View, CA), and hard copies. Data was analyzed by IBM SPSS Statistics, version 20 (IBM Corp., Armonk, NY) and submitted responses were represented in terms of percentage and frequency.

Results: Among the participants, 76.61% (n=167) showed their familiarity with simulation and VR, and 54.50% (n=119) reported limited realism and high cost as the challenges for implementing simulation and VR. All participants (100%) reported the lack of availability of simulation and VR in their institutions. Among the participants, 81.80% (n=201) confirmed the improvement of surgical skills, decreased errors, and increased performance by using simulation and VR. Spearman’s rank-order correlation showed a positive relationship (r_s_=0.159) and a statistically significant result (p=0.019).

Conclusion: Despite the challenges and limitations, the integration of simulation and VR into the training curriculum should be prioritized with the allocation of funds and training of supervisors. Competencies of the surgical trainees should be signed off including specified time on rehearsal before practical implications.

## Introduction

The use of virtual simulation (VS)-based institutional practices is increasing because of the development of computer-based technologies. For reality-based educational approaches in healthcare, the use of simulation is on the rise, especially in the field of undergraduate education. Previous studies have shown that for learners, simulation and reality-based learning help incorporate theoretical knowledge into the skills required for their independent practice [1]. The simulation is defined as “a process that involves making of a hypothetical environment with the incorporation of actual reality, student participation, and complex learning processes, which support feedback, evaluation, and reflection” [2].

Terms such as VS, virtual reality (VR), and augmented reality (AR) are often used interchangeably, though they have distinct definitions [3]. Simulation and VR have introduced significant changes in medical education and healthcare settings. They have helped surgeons understand the complexity of different surgical procedures [4].

There has been a gradual shift in the incorporation of the latest technology such as VR and artificial intelligence (AI) in medical education, with De Ponti et al. having shown a positive impact of VR among students in medical education [5-7]. Therefore, the integration of VR is required in the curriculum to increase its acceptance. VR has also shown that surgical trainees can practice hands-on skills on virtual patients which can increase their skills while keeping the chances of errors very low [5-7].

Previous studies have explored the perception, knowledge, and awareness of simulation, VR, and AI among healthcare professionals in Pakistan. However, none of them focuses on the perception, use, and integration of these among surgical specialties in the southern Punjab region of Pakistan [4,6].

To understand the effect of simulation and VR on medical education, their contribution towards enhancing the skills of surgical trainees, and making them capable of keeping pace with advanced procedures, a high-quality research study was required [4].

The objectives of our study were to evaluate the perception of simulation and VR among surgeons and to identify how the integration of simulation and VR would affect surgical skills and overall performance at public sector tertiary care teaching hospitals in southern Punjab, Pakistan. It also aimed to update the relevant stakeholders about the significance and acceptance of simulation and VR among healthcare professionals in surgery.

## Materials and methods

Our cross-sectional survey was performed from January 1, 2025, to January 31, 2025, at Sheikh Zayed Medical College/Hospital, Rahim Yar Khan, Punjab, Pakistan. Permission was taken from the ethical/institutional review board of Sheikh Zayed Medical College and Hospital, Rahim Yar Khan, Punjab, Pakistan (Ref. No. 101/IRB/SZMC/SZH).

The sample size of 218 was calculated using Raosoft (Raosoft, Inc., Seattle, WA), with a 5% margin of error and an 80% confidence interval. A non-probability convenient sampling technique was used to fill in the pre-designed questionnaire through Google Forms (Google LLC, Mountain View, CA), and hard copies. The data was collected from all four public sector tertiary care teaching hospitals (Nishtar Medical University, Multan; Bahawal Victoria Hospital, Bahawalpur; Allama Iqbal Teaching Hospital, Dera Ghazi Khan; and Sheikh Zayed Medical College and Hospital, Rahim Yar Khan) of southern Punjab, Pakistan.

House surgeons, surgical training fellows, senior registrars, and professors from all four public sector tertiary care teaching hospitals in southern Punjab, Pakistan, who volunteered and provided consent, were included in the study. Medical students and those house surgeons, surgical training fellows, senior registrars, and professors who did volunteer, and did not give consent, were excluded from the study.

The questionnaire was divided into four sections, such as "Section 1. Participants’ Information (4 questions)," "Section 2. Knowledge and Perception (3 questions)," "Section 3. Familiarity with Simulation and VR (2 questions)," and "Section 4. Effectiveness of Simulation and VR in Surgery Curriculum and Real-life Scenarios (6 questions)." Data was entered and analyzed by IBM SPSS Statistics, version 20 (IBM Corp., Armonk, NY). Descriptive statistics, in terms of percentage and frequency, were represented in tables.

Spearman’s rank-order correlation (non-parametric correlation) was run to determine the relationship between the effectiveness of simulation and VR in improving skills compared to conventional methods and the confidence gained for performing procedures if they were previously practiced in simulation and VR.

## Results

Our study involved 218 participants, of which 59.6% (n=130) were male individuals, and 40.4% (n=88) were female individuals. The hospital affiliation of participants, their current position, and years of experience in the field of surgery at the time of filling out the questionnaire are represented in Table 1.

**Table 1:** Participants’ information (n=218), in terms of percentage, %, and frequency, n.

Contents	Frequency (n)	Percentage (%)
Gender		
Male	130	59.6%
Female	88	40.4%
Hospital Affiliation		
Bahawal Victoria Hospital, Bahawalpur	49	22.50%
Nishtar Medical University, Multan	42	19.30%
Sheikh Zayed Hospital, Rahim Yar Khan	93	42.70%
Allama Iqbal Teaching Hospital, Dera Ghazi Khan	34	15.60%
Current Position		
House Surgeon	47	21.6%
Surgical Training Fellow	124	56.9%
Senior Registrar	32	14.7%
Professor	15	6.9%
Experience in Surgery		
0-2 years	110	50.5%
3-5 years	64	29.4%
6-10 years	24	11.0%
>10 years	20	9.2%

Regarding knowledge and perception of simulation and VR, 67.40% (n=147) of participants reported never having used simulation or VR in their surgical practice. Meanwhile, 72.70% (n=158) highlighted decreased errors and improved performance as benefits of simulation and VR in complex procedures. Among the participants, 81.80% (n=201) highlighted the importance of simulation and VR in improving surgical skills. Also, 100% (n=218) of the participants confirmed the lack of availability, and 54.50% (n=119) believed that limited realism of simulation and VR, and high cost, are challenges for the implementation of simulation and VR in tertiary care teaching hospitals of southern Punjab, Pakistan (Table 2).

**Table 2:** Knowledge and perception of participants’ with simulation and VR, in terms of percentage, %. VR, virtual reality.

Variable	Response	Percentage (%)
Have you ever used simulation and VR in your surgical practice?	Yes	32.60%
	No	67.40%
What do you think are the benefits of using simulation and VR in surgery?	Improvement in surgical skills	81.80%
	Decreased errors during surgery	72.70%
	Improved understanding of anatomy	45.50%
	Increased decision-making and critical thinking	36.40%
	Increased confidence in performing complex procedures	72.70%
	Ease to practice rare and high-risk procedures	54.50%
	None	
What do you think are challenges of using simulation and VR in surgery?	High cost	54.50%
	Lack of availability in your institution	100%
	Limited realism of simulation and VR	54.50%
	No standardized protocols in surgical training	36.40%
	Time-consuming to be used to the technology	9.10%
	Limited training for specific surgeries	18.20%

About familiarity with simulation and VR, 76.61% (n=167) of participants showed their familiarity. However, only 12.54% (n=27) were using laparoscopic simulators, with only 2.75% (n=6) selecting the option of using robotic simulators in their surgical practice (Table 3).

**Table 3:** Familiarity with simulation and VR, in terms of percentage, %. VR, virtual reality.

Variable	Response	Percentage (%)
How familiar are you with simulation and VR in surgery?	Very much	19.27%
	Somewhat	57.34%
	Not at all	23.39%
What types of simulations and VR have you used in your surgical practice?	Laparoscopic simulator	12.54%
	Robotic simulators	2.75%
	Virtual reality-based dissection	2.75%
	Endoscopic/colonoscopy simulator	5.27%
	3D/4D imaging-based simulation	2.75%
	Other	6.54%
	None	67.40%

The effectiveness of simulation and VR was also assessed on the Likert scale, with the most effective to very less effective. Although 76.61% (n=167) of our respondents were familiar with simulation and VR in surgery, 97.2% (n=210) of them never used simulation or VR as a part of their training. Of the participants, 90.8% (n= 198) believed that they would be confident in performing the procedures if they had practiced them previously on simulation and VR. When asked about the importance of the implications of simulation and VR in surgery, 100% (n=218) of the participants strained on the importance of its implementation into the surgical residency curriculum. Non-availability, lack of infrastructure, and reluctance to change in traditional ways were the barriers to their implementation. Of the participants, 62.4% (n=136) suggested the combination of hands-on and simulation-based training in their surgical training curriculum (Table 4).

**Table 4:** Effectiveness of simulation and VR in surgery curriculum and real life scenarios, in terms of percentage, %. VR, virtual reality.

Variable	Response	Percentage (%)
How would you rate the effectiveness of simulation and VR in improving skills compared to conventional methods (e.g., cadaveric dissection, live surgeries)?	Most effective	49.5%
	Somewhat effective	50.5%
	Same	0%
	Less effective	0%
	Very less effective	0%
Have you used simulation or VR as part of your surgical training?	Frequently	2.8%
	Occasionally	0%
	Rarely	0%
	Never	97.2%
Will you be confident in performing procedures if you have practiced them on simulation or VR?	Very confident	45.4%
	Confident	45.4%
	Neutral	0%
	Not confident	9.2%
	Not confident at all	0%
What’s the importance of simulation and VR into the surgical residency curriculum?	Very important	41.7%
	Important	58.3 %
	Neutral	0%
	Not important	0%
	Not important at all	0%
Would you prefer simulation and VR-based training or more hands-on training in your surgical practice?	More simulation & VR-based training	11%
	More hands-on training	17.4 %
	Combination of both	62.4%
	No preference	9.2%
What are the barriers to the implementation of simulation and VR in your institution?	Lack of funding	72.70%
	Lack of infrastructure	81.80%
	Lack of faculty for use of simulation & VR	54.50%
	Reluctance to change in traditional ways	36.40%

Spearman’s rank-order correlation showed a positive correlation between the effectiveness of simulation and VR in improving skills compared to conventional methods and the confidence in performing procedures if practiced in simulation or VR. Although, Spearman's correlation was weakly positive (Spearman's rho=0.159), a P-value of 0.019 confirmed this as statistically significant (Table 5).

**Table 5:** Spearman’s rank-order correlation (non-parametric correlation). * Correlation is significant at the 0.05 level (2-tailed). VR, virtual reality.

			How would you rate the effectiveness of simulation and VR in improving skills compared to conventional methods (e.g. cadaveric dissection, live surgeries)?	Will you be confident in performing procedures if you have practiced them in simulation or VR?
Spearman's rho	How would you rate the effectiveness of simulation and VR in improving skills compared to conventional methods (e.g. cadaveric dissection, live surgeries)?	Correlation Coefficient	1.000	.159^*^
		Sig. (2-tailed)	.	.019
		N	218	218
	Will you be confident in performing procedures if you have practiced them in simulation or VR?	Correlation Coefficient	.159^*^	1.000
		Sig. (2-tailed)	.019	.
		N	218	218

## Discussion

Surgical trainees have always been under restrictive environments to learn essential skills needed for their career progression [3]. The use of simulation-based digital learning platforms has been beneficial in reducing the cost of learning practical skills. Previous studies have also shown conclusive evidence of the effectiveness of the integration of simulation in medical education. Though the implication of simulation and VR has been widely used for intravenous cannulation, catheterization, and imaging, its use in the field of surgery has been limited [1].

Laparoscopic suturing is considered one of the most difficult skills to learn in surgery. A literature review conducted in 2014 on the use of simulation for the acquisition of suturing skills in laparoscopy showed conclusive evidence for the training of surgical residents [8]. It also showed that the skills learned on simulation were not only learned but also transferred onto the operating table [8].

One of the major challenges in surgery is the frequency of errors, which can greatly be reduced by simulation-based practice. Complex anatomical dissections during simulation and VR followed by automatic feedback of the rehearsal, have demonstrated a decrease in the number of errors during practical scenarios since the reporting of the first VR surgical simulator by Satava in 1993 [5,6,8-10].

Although 67.4% (n=147) of participants reported never having used simulation or VR in their surgical practice, all participants (100%, n=218) acknowledged the importance, effectiveness, and potential integration of simulation and VR into the training curriculum. Barriers to the implementation of simulation and VR in Pakistan include limited awareness, adherence to conventional methods, and a lack of resources [6]. Another cross-sectional study in Pakistan also showed limited availability and access to the implementation of simulation-based learning [11].

A systemic review concluded that the use of both simulation and VR-based training and conventional cadaveric dissections not only helped the surgeons handle intraoperative complications and anatomical variations but also met the educational needs of trainees [10]. The use of simulators was also proved to be cost-effective, with the benefit of being used multiple times, and adjusting the circumstances according to the difficulty level [12]. This also improved the decision-making, critical thinking, and confidence of the trainees [2].

There are currently different simulation-based learning programs, which create an environment and rehearsal platform for teamwork, and for work under high-pressure situations. For trainees, these types of devices can also be used by examiners to assess the skill level and set the difficulty level according to the exam needs. Just like previous studies in Pakistan, our study also showed the lack of funding, infrastructure, faculty, the reluctance to counter conventional methods, and non-availability as the challenges for implementing simulation and VR in the public sector, tertiary care teaching hospitals of southern Punjab, Pakistan [3,4,6].

The confounding variables in our study, such as the position of the participants and their experience did not seem to influence the benefits, effectiveness, or importance of incorporating simulation and VR into the surgical training curriculum. Seymour reported in an observational study that was focused on surgical residents, the importance of simulation and VR in improving surgical performance in real-life scenarios, and reducing the time for overall procedure [10].

Despite the challenges for the implementation of simulation and VR into the surgical training, stakeholders, and mentors within the healthcare community can develop a program for local production, that can greatly reduce the expense within the limited available resources. This can also greatly reduce the time spent on training the trainees, and the effect of firewalls [3].

Limitations

Non-probability convenient sampling technique was used for data collection, which can have its own disadvantages such as sampling bias and lack of generalizability. also, the participants who were most frequently using social media, and other online platforms were most easily accessible for data collection for our study.

## Conclusions

The insights gained from this study highlight the benefits of simulation and VR in surgical practice, guiding stakeholders in integrating these technologies into the surgical curriculum. Despite financial challenges and limitations, their integration into the training curriculum should be prioritized. Funds should be allocated for the acquisition of simulation and VR devices, and supervisors should be trained in their use. The competencies of the surgical trainees should also be signed off by supervisors, which must include a specified amount of time spent rehearsing with simulation and VR devices before practical application.

## References

[1] Wu Q, Wang Y, Lu L, Chen Y, Long H, Wang J (2022). Virtual simulation in undergraduate medical education: a scoping review of recent practice. Front Med (Lausanne).

[2] Kaldheim HK, Bergland Å, Ølnes MA (2019). Use of simulation-based learning among perioperative nurses and students: a scoping review. Nurse Educ Today.

[3] McGrath JL, Taekman JM, Dev P (2018). Using virtual reality simulation environments to assess competence for emergency medicine learners. Acad Emerg Med.

[4] Khan Z, Adil T, Oduoye MO, Khan BS, Ayyazuddin M (2024). Assessing the knowledge, attitude and perception of extended reality (XR) technology in Pakistan's healthcare community in an era of artificial intelligence. Front Med (Lausanne).

[5] Mergen M, Meyerheim M, Graf N (2023). Reviewing the current state of virtual reality integration in medical education - a scoping review protocol. Syst Rev.

[6] Mehmood Qadri H, Bashir M, Khan M (2024). Knowledge, awareness and practice of artificial intelligence and types of realities among healthcare professionals: a nationwide survey from Pakistan. Cureus.

[7] De Ponti R, Marazzato J, Maresca AM, Rovera F, Carcano G, Ferrario MM (2020). Pre-graduation medical training including virtual reality during COVID-19 pandemic: a report on students' perception. BMC Med Educ.

[8] Dehabadi M, Fernando B, Berlingieri P (2014). The use of simulation in the acquisition of laparoscopic suturing skills. Int J Surg.

[9] Satava RM (1993). Virtual reality surgical simulator. The first steps. Surg Endosc.

[10] Seymour NE (2005). Integrating simulation into a busy residency program. Minim Invasive Ther Allied Technol.

[11] Khalid T, Yaqoob H, Syed FA, Kazmi SM (2024). Assessing availability and trainees' perceptions of simulation and augmented reality in prosthodontics postgraduate education in Pakistan: a cross-sectional study. BMC Med Educ.

[12] Chahal B, Aydin A, Ahmed K (2024). Virtual reality vs. physical models in surgical skills training. An update of the evidence. Curr Opin Urol.

